# Antibiotic Prescription Patterns in the Paediatric Primary Care Setting before and after the COVID-19 Pandemic in Italy: An Analysis Using the AWaRe Metrics

**DOI:** 10.3390/antibiotics11040457

**Published:** 2022-03-29

**Authors:** Elisa Barbieri, Cecilia Liberati, Anna Cantarutti, Costanza Di Chiara, Angela Lupattelli, Michael Sharland, Carlo Giaquinto, Yingfen Hsia, Daniele Doná

**Affiliations:** 1 Division of Paediatric Infectious Diseases, Department for Women’s and Children’s Health, University of Padua, 35100 Padua, Italy; cecilia.liberati@unipd.it (C.L.); costanza.dichiara@phd.unipd.it (C.D.C.); carlo.giaquinto@unipd.it (C.G.); daniele.dona@unipd.it (D.D.); 2 Unit of Biostatistics, Epidemiology and Public Health, Department of Statistics and Quantitative Methods, University of Milano-Bicocca, 20126 Milan, Italy; anna.cantarutti@unimib.it; 3 National Centre for Healthcare Research and Pharmacoepidemiology, Department of Statistics and Quantitative, Methods, University of Milano-Bicocca, 20126 Milan, Italy; 4 Società Servizi Telematici—Pedianet, 35100 Padua, Italy; 5 PharmacoEpidemiology and Drug Safety Research Group, Department of Pharmacy, and PharmaTox Strategic Research Initiative, University of Oslo, 0316 Oslo, Norway; angela.lupattelli@farmasi.uio.no; 6 Centre for Neonatal and Paediatric Infection, St George’s University of London, London SW17 0RE, UK; msharland@sgul.ac.uk (M.S.); y.hsia@qub.ac.uk (Y.H.); 7 School of Pharmacy, Queen’s University Belfast, Belfast BT9 7BL, UK

**Keywords:** antibiotics, AWaRe classification, COVID-19 pandemic

## Abstract

The containment measures following COVID-19 pandemic drastically reduced airway infections, but they also limited the access of patients to healthcare services. We aimed to assess the antibiotic prescription patterns in the Italian paediatric primary care setting before and after the containment measures implementation. For this retrospective analysis, we used a population database, Pedianet, collecting data of patients aged 0–14 years enrolled with family paediatricians (FP) from March 2019 to March 2021. Antibiotic prescriptions were classified according to WHO AWaRe classification. An interrupted time series evaluating the impact of the containment measures implementation on the monthly antibiotic index, on the access to watch index, and on the amoxicillin to co-amoxiclav index stratified by diagnosis was performed. Overall, 121,304 antibiotic prescriptions were retrieved from 134 FP, for a total of 162,260 children. From March 2020, the antibiotic index dropped by more than 80% for respiratory infections. The Access to Watch trend did not change after the containment measures, reflecting the propensity to prescribe more broad-spectrum antibiotics for respiratory infections even during the pandemic. Similarly, co-amoxiclav was prescribed more often than amoxicillin alone for all the diagnoses, with a significant variation in the trend slope for upper respiratory tract infections prescriptions.

## 1. Introduction

Antibiotics are among the most prescribed medicines worldwide, especially in pre-school-aged children [1]. Outpatient antibiotics prescriptions account for most of the total antimicrobials use in the general paediatric population, and its trend is influenced by seasons. This tendency follows the incidence of respiratory infections, especially community-acquired pneumonia (CAP); acute otitis media (AOM); and pharyngitis, the most common reason for antibiotics prescription in paediatrics [2]. Over-prescribing and inappropriate prescribing increases antimicrobial resistance and results in preventable adverse drug reactions [3,4]. Before the coronavirus disease 2019 (COVID-19) pandemic, paediatric outpatient prescription data in Italy showed limited use of narrow-spectrum antibiotics for CAP [5], as well as a tendency to prescribe broad-spectrum antibiotics for pharyngitis. Moreover, early antibiotic prescription behaviour for AOM was described against the “wait and see” recommended strategy [6,7].

Since the beginning of the COVID-19 pandemic in March 2020, the implementation of containment measures (especially school’s closure) drastically reduced the incidence of respiratory tract infections in the cold season globally. Consequently, differently from prescriptions for chronic diseases medication [8], a reduction in overall antibiotic prescription was observed in the first months of the pandemic, compared to the same months of the previous season [9,10,11]. On the other hand, the modified procedures of access to medical services and the implementation of remote consultations (by email or phone in place of in-person visits) might have changed the antibiotic prescribing behaviour, possibly varying the use of broad-spectrum antibiotics in spite of narrow-spectrum ones.

This study aimed to assess the impact of COVID-19 containment measure implementation on antibiotic prescriptions in the Italian paediatric primary care setting. To have our results more generalisable and comparable with data from other countries, we used the AWaRe classifications and metrics developed in 2017 by the WHO Expert Committee on Selection and Use of Essential Medicines (and further updated) [12], as well as other metrics specific for the paediatric population [13]. AWaRe classifies the antibiotics into three categories on the basis of the empiric treatment: Access (first-choice and second-choice antibiotics that are generally narrow spectrum with less resistance potential—e.g., amoxicillin and co-amoxiclav), Watch (antibiotic classes with broader spectrum and hence resistance potential—e.g., fluoroquinolones, macrolides, and second- and third-generation cephalosporins), and Reserve (last-resort option antibiotic classes).

## 2. Results

### 2.1. Overall Results

**Figure 1:**
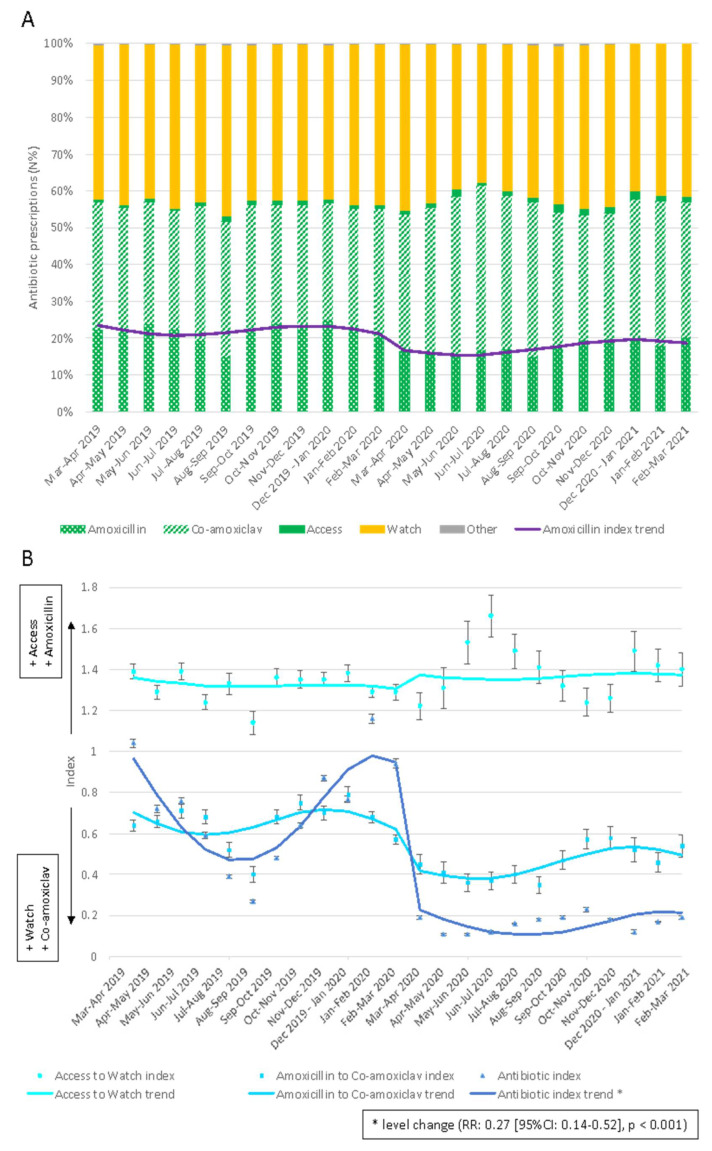
Monthly antibiotic prescriptions in children 0–14 years of age in Italy in the 12 months before and after the COVID-19 containment measures implementation (**A**) and interrupted time series of the monthly index (**B**) along with the 95% CI. Pedianet 2019–2021.

Overall, 121,304 antibiotics were prescribed from 134 family paediatricians (FPs) to 162,260 children, with 278,909 person-years of follow-up during the study period. Half of the patients were males (51.9%), and almost 60% of the children resided in the North of Italy (Piemonte, Lombardy, Friuli Ven.-Giulia, and Veneto regions). The mean age of the population in the period before the COVID-19 containment measure implementation was 6.2 years, while in the period after, it was 6.4 years (Table S1 in the Supplementary Materials). The monthly Access prescription rates were around 60% in both pre- and post-COVID-19 containment measure implementation periods, with co-amoxiclav accounting for most prescriptions. Co-amoxiclav prescription rates varied from a minimum of 32% between October and November 2019 and between December 2019 and January 2020 to a maximum of 45% between June and July 2020 (Figure 1 and Table S2 in the Supplementary Materials).

The antibiotic index decreased by 80% (relative rate (RR) 0.27 (95%CI: 0.15–0.46), *p* < 0.001) following the containment measures implementation in March 2020 from a maximum of 1.2 antibiotics per child in the pre-period to a maximum of 0.23 antibiotics per child in the post-period. The increase in the Access to Watch index in the months immediately after March 2020 (from 1.22 to 1.66 prescriptions per child) did not reach statistical significance (Figure 1 and Table S1 in the Supplementary Materials).

The amoxicillin to co-amoxiclav index decreased in the months after the implementation of containment measures, following the increase in the co-amoxiclav prescription rate. However, it was not significant. The peak was registered between June and July, with 1.66 Access prescriptions per every Watch prescription (Figure 1 and Table S2 in the Supplementary Materials).

The number of telemedicine consultations increased significantly by five times following the containment measures implementation, with a change in the trend. On the contrary, the number of well-child visits decreased significantly by 80% from March 2020 (Figure S1 in the Supplementary Materials).

### 2.2. Results by Diagnosis Class

In the study period, 20,357 (16.8%) antibiotics were prescribed for a diagnosis of lower respiratory tract infection (LRTI), 3704 (3.1%) for skin and soft tissue infection (SSTI), 58,793 (48.5%) for upper respiratory tract infection (URTI), and 2302 (1.9%) for urinary infection (UTI). Before the containment measures implementation, the diagnosis most frequently associated with an antibiotic prescription was URTI, while in the post-implementation period, especially after August 2020, most of the prescriptions were not associated with a diagnosis (from a minimum of 33.1% in March 2020 to a maximum of 58.8% in December 2020 of prescriptions prescribed without a diagnosis) (Table S3 in the Supplementary Materials). 

**Figure 2:**
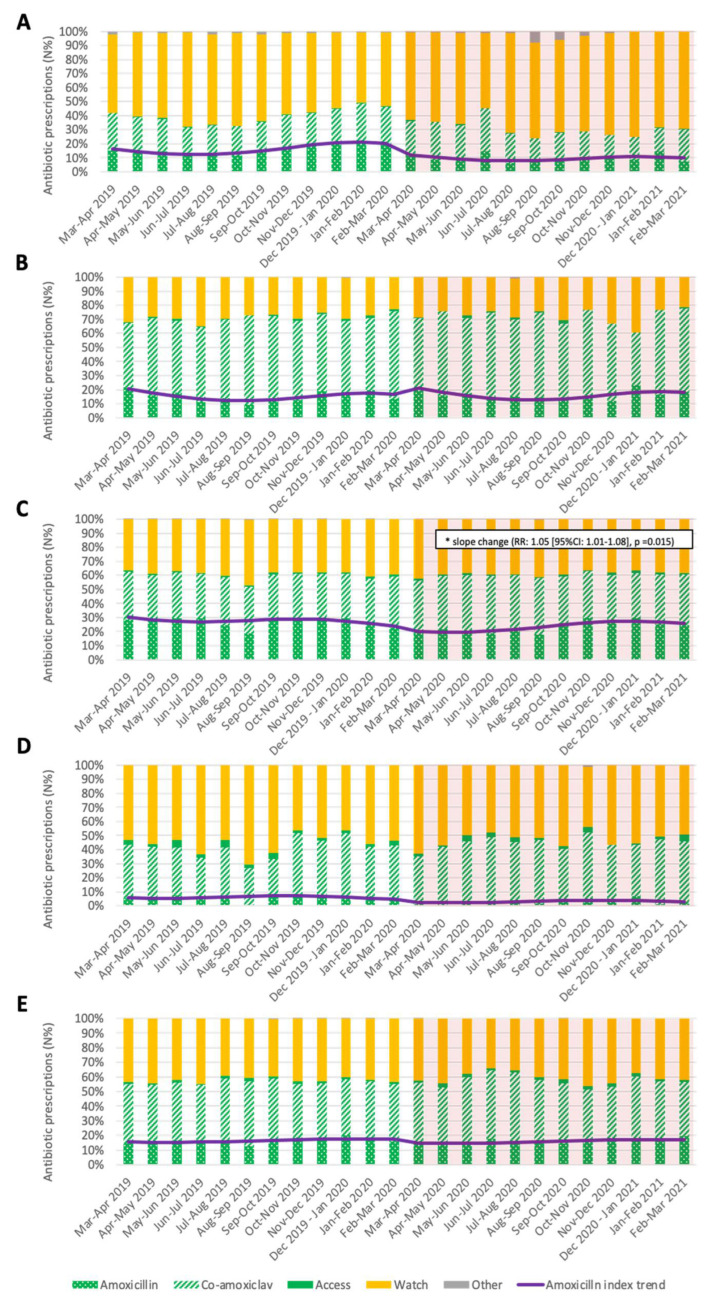
Monthly antibiotic prescriptions in children 0–14 years of age in Italy in the 12 months before and after COVID-19 containment measures implementation by diagnosis class ((**A**) LRTI, (**B**) SSTI, (**C**) URTI, (**D**) UTI, (**E**) NA). Pedianet 2019–2021.

The monthly Access prescriptions rate for LRTI, constituted mainly by co-amoxiclav, was above the 35% before the COVID-19 containment measures implementation, while in the post-period was below 30% (Figure 2 and Table S4 in the Supplementary Materials). 

The monthly Access rate for SSTI and URTI was higher (around 70% and 60%, respectively) than LRTI rates, and co-amoxiclav rates accounted for 70% and 60% of Access prescriptions, respectively (Figure 2 and Table S4 in the Supplementary Materials).

Regarding UTI, the Watch rate was above 50% for most of the months, and among Access prescriptions, co-amoxiclav was the most prescribed antibiotic, accounting for around 90% of the Access prescriptions (Figure 2 and Table S4 in the Supplementary Materials).

The amoxicillin index trend did not vary in level after implementing COVID-19 containment measures for any of the diagnosis classes; however, the trend slope for URTI varied significantly (Figure 2 and Table S4 in the Supplementary Materials).

**Figure 3:**
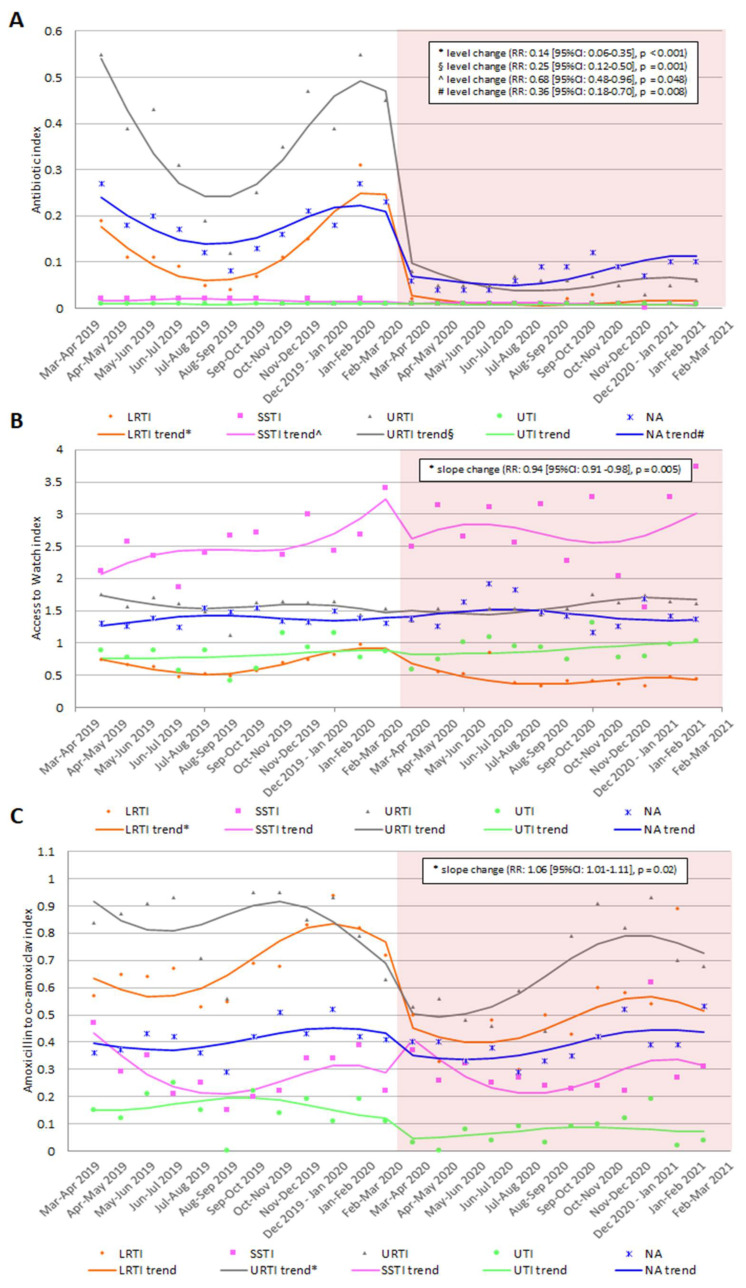
Antibiotic index (**A**), Access to Watch index (**B**), and amoxicillin to co-amoxiclav index (**C**) in children 0–14 years of age in Italy in the 12 months before and after COVID-19 containment measures implementation by diagnosis class. Pedianet 2019–2021.

The number of antibiotics prescribed for a specific diagnosis class per child decreased significantly after implementing the containment measures for all the diagnoses except for UTIs (Figure 3 and Table S4 in the Supplementary Materials). The highest fall in the trend level was observed for LRTIs (−86%), followed by URTIs (−75%) and SSTIs (−32%). Before the COVID-19 containment measures, the antibiotic index for NA class was around 0.2, while after the implementation, the trend decreased immediately by 64% (Figure 3 and Table S4 in the Supplementary Materials).

As regarding the Access to Watch trend, the index was above two just for SSTIs prescriptions. The lowest index was observed for LRTIs in November 2020 (0.33 Access antibiotics prescribed for every Watch), where it was observed the only variation in the trend slope after the COVID-19 containment measures implementation (Figure 3 and Table S4 in the Supplementary Materials).

Co-amoxiclav was prescribed more often than amoxicillin alone for all the diagnoses, with no variation in the trend after the containment measures implementation, but a variation in the trend slope for URTI prescriptions (Figure 3 and Table S4 in the Supplementary Materials).

## 3. Discussion

Our study showed an immediate 80% decrease in the overall monthly antibiotic prescriptions per child, but no variation was noticed in the Access to Watch index and co-amoxiclav to amoxicillin index. In general, the decrease was noticed for all diagnoses except for UTIs. Still, the Watch monthly antibiotic prescription rates remained higher than 30% for most of the respiratory tract infection.

To our knowledge, this is the first study assessing antibiotic prescription patterns before and after the COVID-19 pandemic according to the AWaRe classifications in the paediatric outpatient population in Italy. Providing information from a national electronic Healthcare Record database allowed us to have a likely picture of antibiotic prescriptions and infections trend (by proxy) and to explore variations in prescribing appropriateness, using as indicators the antibiotic index and the ratio of amoxicillin on co-amoxiclav, in line with national indicators [1]. The Access to Watch Index trend was also assessed, according to 2017 WHO classification, which listed the antibiotics as Access, Watch, and Reserve on the basis of recommendations for their practical use for the most common infections [14]. In the 13th General Programme of Work 2019–2023, the WHO proposed a country-level target of at least 60% of total antibiotic consumption being Access group antibiotics.

The results of our study are consistent with previous findings in the general and paediatric populations in other countries, including the USA, South Korea, Canada, Finland, and the UK. The number of antibiotics prescribed per child decreased after the COVID-19 containment measures implementation in March 2020 by 80%, and the fall was more evident for respiratory tract infections (URTIs and LRTIs), the classes for which were prescribed most of the antibiotics. In general, the reduction in antibiotic prescriptions follows the decline in primary care and ED visits related to infections, especially acute respiratory infections (ARIs) [15,16,17,18]. The containment measures implementation effectively reduced the transmission of COVID-19 and the spread of other respiratory viruses, such as influenza virus [19,20] and respiratory syncytial virus [21,22], which used to be the leading causes for ARI hospitalisations and ED admission every year. Likewise, other viral illnesses showed a downward trend after the pandemic begin [23,24]. It seems that this effect lasted in the 12 months after the containment measures implementation.

In our study, overall Access and Watch antibiotics prescriptions fell just after the beginning of the COVID-19 pandemic in March 2020. In the same way, a national report from Portugal analysed the overall antibiotic prescriptions in the general population in the outpatient setting after the pandemic outbreak, finding a significant reduction in the Watch group antibiotics; this is explained by the authors as a possible result of the decrease of infections with the containment measures and cancellation of appointments [9]. On the opposite side, a survey in Jordan highlighted an increase in the use of Watch antibiotics by 26% from 2019 to 2020 and a reduction in amoxicillin use [25]; however, this study involved the collection of national antimicrobial consumption data in hospitals and community pharmacies, with a different design considering parenteral antibiotic use in hospitalised patients that probably increased during the pandemic, as suggested by the authors.

To better describe the antibiotic prescription drivers in our country, we stratified the data according to the diagnosis, adopting four categories. Overall, in our report, prescriptions for RTIs dropped significantly; on the contrary, the antibiotic prescription rate for UTIs did not change after the pandemic began, reflecting the different aetiologies and pathogeneses, not being related to interpersonal transmission. Differently from our findings, a study using general practice data in the Netherlands showed a reduction in episodes treated with antibiotics both for ARIs and UTIs in adults after implementing anti-covid measures. Authors postulated a general attitude to avoid medical visits for uncomplicated infections during the pandemic [11].

Interestingly, oppositely to the reduction in the trend of infections and overall prescriptions, antibiotic prescription appropriateness did not improve the same way. Access antibiotics should always be the first-line therapy, with amoxicillin for URTIs and LRTIs and co-amoxiclav for UTIs and SSTIs. In our study, the ratio of Watch on Access prescriptions, as well as the ratio of amoxicillin on co-amoxiclav, did not change after the containment measures implementation, as would be expected, assuming that the overall incidence of severe or non-responding to first-line treatment infections is dramatically decreased [26]. As previously noted in other studies, broad-spectrum antibiotics remain widely prescribed in primary care in Italy, and from the current study, it is evident that this did not improve after the COVID-19 pandemic [27].

Amoxicillin to co-amoxiclav index is considered a strong indicator of antibiotic prescription appropriateness in the context of antimicrobial surveillance in the outpatient setting. The amoxicillin to co-amoxiclav index should be used by regulators as a proxy to measure prescribing appropriateness in the general outpatient population and as a stewardship metric. It is not indicated to measure the appropriateness of a single diagnosis class. Amoxicillin is recommended as a first-line therapy for different respiratory tract infections (including AOM, pharyngitis, and CAP); however, the treatment of choice can vary on the basis of the child’s age, history of immunisation, comorbidities, and allergies. In 2016, the European Commission indicated a value of >=4 as the target ratio to achieve an appropriate control of broad-spectrum prescriptions [28]. Our report confirms previous findings reporting the level of this index to be below the threshold. In our report, the index was below 1 before and after March 2020. 

Aware indicators were for the first time used in an international report analysing the 2015 consumption data of oral antibiotic formulations for children from a global database [13]. On average, Access to Watch Index was 6. However, in Italy, the value was reported to be 2.5, suggesting a consumption of Watch antibiotics that is more than double than the average. However, these values cannot be ultimately compared to the present study for the methodological differences. While in the first case, sales were counted, we obtained the prescriptions data that constitute a different surrogate of the effective administration to children. Furthermore, in the 2015 report, co-amoxiclav was categorised in the Watch group, while from 2019, it entered the Access one [12].

Amoxicillin Index and Access to Watch Index were stratified according to the diagnostic categories in our report. Independently from the spread of COVID-19, in our research, the most critical point remains the treatment of LRTI, for which Watch antibiotics (including macrolides) are the most prevalent. From a recent WHO survey, the use of macrolides is reported to be increased after the COVID-19 pandemic with around half of the countries included reporting a rise in azithromycin prescription for patients with respiratory-like symptoms in healthcare facilities [29].

Strengthening empirical therapy for CAP guidelines diffusion is urgent in our outpatient setting in order to improve prescribing behaviour and reduce the inappropriate broad-spectrum prescriptions (i.e., the combination of amoxicillin–macrolide or macrolides alone as treatment strategy for CAP) [30]. Indeed, the increased incidence in *Streptococcus pneumoniae* resistance to macrolides over the past two decades, up to 30% in Europe and 30–35% in USA [31], is a matter of concern. Notably, the resistance rate of *S. pneumoniae* to penicillin decreased after the introduction of universal infant immunisation with the pneumococcal conjugate vaccine and was estimated to be about 12% in 2019 in Europe [32]. Still, second-generation cephalosporin (which belong to the Watch category) prescriptions for LRTIs, which should be considered in case of treatment failure in younger children, contributed to the increased prescription of Watch antibiotics for both LRTI and URTI in our study.

A study from the UK reported stratified antibiotic prescriptions for CAP in the paediatric population before and after the COVID-19 pandemic: for children up to four years, overall amoxicillin prescription rate per 1000 population decreased from March 2020 by 62.5% [33]. However, other diagnoses were not analysed, and access to watch index and ratio of amoxicillin to co-amoxiclav were not studied.

In our report, Access prescription rates for URTI were high. However, co-amoxiclav was still predominantly prescribed, especially just after March 2020, determining a variation in the amoxicillin to co-amoxiclav slope. It should be addressed that most URTIs have a viral aetiology, and if bacterial, *S. pyogenes* represents the leading cause of pharyngitis, while *S. pneumoniae* is one of the leading causes of acute otitis media (AOM). Amoxicillin alone should still be considered the first choice in most of the cases.

We can speculate that the lack of specific surveillance data on antibiotic resistance in primary care, particularly after the COVID-19 pandemic, directly impacts prescribers’ behaviour, which might prefer antibiotics with a higher coverage, although this is not supported by evidence and guidelines.

After the containment measures implementation, telemedicine was employed worldwide in different fields and situations [34,35,36]. A reduction in face-to-face consultations for ill children emerged in our analysis: parents and paediatricians adopted phone calls and email more often to address health issues and respiratory symptoms with fever, which could have led to a greater diagnostic uncertainty and a precautional prescription of broad-spectrum antibiotics. This hypothesis can be confirmed by the drastic increase in the number of prescriptions not associated with a formulated diagnosis after March 2020. Indeed, even before the pandemic, children with presumed ARIs or conjunctivitis were more likely to receive antibiotics when undergoing telemedicine visits compared to other settings (urgency attendance, family paediatricians) [37,38]. However, evidence from a systematic review analysing the impact of remote consultation in primary care on antibiotic prescription before the COVID-19 pandemic were inconclusive [39].

Antimicrobial stewardship (AMS) in the outpatient setting is a well-known topic that needs to be addressed and implemented, as family paediatricians’ prescriptions represent the majority of antibiotics dispensed to the paediatric population. During the pandemic, AMS programs had to cope with several problems, as reported from a WHO survey: decreased in national and local funding for antimicrobial resistance (AMR) surveillance activities (more frequently reported in low- and middle-income countries), decreased ability to work in partnerships, reduction in the staff available for AMR activities, and reduction in laboratory supplies and equipment for AMR [29]. Moreover, factors driving inappropriate antibiotic prescribing in the outpatient setting (such as patient pressure to receive an antibiotic, even when they are not indicated; time constraints limiting accurate treatment plan; diagnostic uncertainty; and externalised responsibility of paediatricians outside themselves) [40] could have been further magnified during the COVID-19 pandemic.

Considering the important impact on AMS on AMR, outpatient AMS implementation should be continued as the pandemic continues. Continuous education and guidelines supplied to family paediatricians may raise antibiotic prescription consciousness and improve prescription appropriateness. The CDC in 2017 outlined the core elements of outpatient AMS. Apart from education and expertise, the other elements include a public commitment for AMS, action for policy and practice (e.g., requiring a written justification for non-recommended antibiotic prescribing), and tracking and reporting systems [41].

The strengths of our study are its size, generalisability, and representative coverage of paediatric patients in Italy. It is particularly relevant because, to the best of our knowledge, there are no other studies assessing antibiotic prescription patterns before and after the COVID-19 pandemic according to the AWaRe classifications in the same population.

Our study has several limitations. First, it is retrospective in nature, and there is significant heterogeneity in how FP reported data on the electronic medical chart, not always following the standardised list of diagnoses codes. Different diagnoses were categorised a posteriori by the authors. Second, we cannot say with certainty that every prescribed medicine was administered. Indeed, some paediatricians might have instructed parents to follow a “wait and see” approach, administering antibiotics only if needed. Third, some antibiotic indications were not considered in the analysis, such as odontoiatric and ophthalmological prescriptions. Fourth, prescribing behaviours among the different family paediatricians vary. However, we included only prescribers participating for the whole study period. Moreover, significant heterogeneity is not properly a limit of our study but a characteristic of the outpatient management, and therefore we can say it is a close portrait of the Italian paediatric primary care situation.

## 4. Materials and Methods

This is a retrospective analysis of antibiotic prescribing from 9 March 2019 to 9 March 2021 using Pedianet, a paediatric primary case database. Pedianet captures data from the routine patient care using JuniorBit^®^ software. This database contains information on patient demographic characteristics, prescriptions, vaccinations, diagnoses, and referrals to specialists and hospitalisations. The Pedianet database contains patient-level data from children followed by around 160 FPs distributed throughout several Italian regions, including Friuli-Venezia Giulia, Liguria, Lombardia, Piemonte, Veneto, Lazio, Marche, Toscana, Abruzzo, Campania, Sardegna, and Sicilia. The Pedianet database has been widely used since 2004 to conduct pharmacoepidemiological and pharmacovigilance studies [27]. According to the Italian NHS, each child is assigned to a FP who is the primary referral for health-related matters. Data are anonymised with a monthly update to a centralised database based in So.Se.Pe. Srl. the legal owner of Pedianet, in Padova, assessing antibiotic prescription patterns before and after the COVID-19 pandemic according to the AWaRe classifications in the paediatric outpatient population in Italy [42]. Only family paediatricians who were part of Pedianet for the whole study period were considered for this study. Due to the study’s retrospective nature and the use of anonymised data, no ethical consent was required to conduct the study. The study protocol and the data access were approved by the Internal Scientific Committee of So.Se.Pe. All children aged <14 years enrolled in the Pedianet database during the study period were included in the analysis. The person-years of children included in the study was calculated from either March 2019 or when patients started receiving care. All patients were following up until the last date of their care or the end of the study period in March 2021.

Antibiotic prescriptions were classified on the basis of Anatomical Therapeutic Chemical Classification (ATC) and route of administration in Access, Watch, and Other according to the Italian Medical competent Authority for Italy (Agenzia Italiana del Farmaco—AIFA) AWaRe classification adapted from the WHO AWaRe classification [1]. 

Diagnoses were retrieved using ICD9-CM codes and classified as follows in line with previously published studies [27] and confirmed by paediatricians specialised in infectious diseases (C.L., D.D): lower respiratory tract infections (LRTI), upper respiratory class infections (URTI), urinary tract infections (UTI), skin and soft tissue infection (SSTI), and not available (NA) (Table S5). Free text algorithms were used to explore reason for prescriptions not associated with an ICD9-CM code.

The primary outcomes were the rates of prescriptions of amoxicillin (ATC: J01CA04), co-amoxiclav (ATC: J01CR02), Access and Watch, the antibiotic index (defined as the number of antibiotic prescriptions per person-years), the Access to Watch index, and the amoxicillin to co-amoxiclav index. The rate of telemedicine consultation and the rate of well-child visits were assessed to account for variation in the use of primary healthcare services.

### Statistical Analysis

Results were summarised using numbers and percentages and stratified by diagnosis class. 

An interrupted time series analysis supposing an abrupt step change and a slope change in monthly outcomes using quasi-Poisson regression models was performed to determine the impact of COVID-19 containment measures implementation [43]. The total number of monthly antibiotic prescriptions, log-transformed person-years, a variable representing the frequency in months (i.e., from 1 to 12), a dummy variable indicating the pre- and post-intervention periods, and a dummy variable indicating the time in months since the intervention sand a variable with Fourier terms to account for seasonality were all the variables considered in the first analysis. For the second analysis, the Watch prescriptions, log-transformed Access prescriptions, and the four other variables previously specified were used. For the third analysis, the co-amoxiclav prescriptions, log-transformed amoxicillin prescriptions, and the four other variables previously specified were used. To explore primary healthcare service variation, the models used the total telemedicine consultations or the total well-child visit, log-transformed person-years, and the four other previously specified variables. Interrupted time series models were performed on the overall data, and then models stratified by diagnosis were used. Autocorrelation was assessed, examining the plot for residuals and the partial autocorrelation function. The corresponding relative risk and 95% confidence interval (95% CI) according to normal approximation were calculated. Two-sided *p* < 0.05 was considered statistically significant. The analysis was performed using R statistical software—v. 4.0.3 (Vienna, Austria) [44].

## 5. Conclusions

These data highlight the tendency in prescribing broad-spectrum antibiotics in the Italian paediatric primary care setting during the pandemic (Watch antibiotics for LRTI and co-amoxiclav for URTI), possibly reflecting the diagnostic uncertainty. Efforts are needed to improve outpatient antimicrobial stewardship programmes, reinforcing the ones already existing and implementing newly available tools for telemedicine. 

## Data Availability

The data used in this study cannot be made available in the manuscript, the supplemental files, or in a public repository due to Italian data protection laws. The anonymised datasets generated during and/or analysed during the current study can be provided on reasonable request from the corresponding author, after written approval by the Internal Scientific Committee (info@pedianet.it).

## Supplementary Materials

The following are available online at https://www.mdpi.com/article/10.3390/antibiotics11040457/s1, Figure S1: Interrupted time series of the monthly index of telemedicine visits (black) and well-child visits (gray) in children 0–14 years of age in Italy in the 12 months before and after COVID-19 containment measures implementation along with 95% CI. Pedianet 2019–2021; Table S1: Demographic characteristics of children included in the analysis in the 12 months before and after COVID-19 containment measures implementation. Pedianet 2019–2021), Table S2: Monthly antibiotic prescriptions in children 0–14 years of age in Italy in the 12 months before and after COVID-19 containment measures implementation. Pedianet 2019–2021), Table S3: Prevalence of. monthly antibiotic prescriptions in children 0–14 years of age in Italy in the 12 months before and after COVID-19 containment measures implementation by diagnosis class on total monthly antibiotic prescriptions. Pedianet 2019–2021, Table S4: Monthly antibiotic prescriptions in children 0–14 years of age in Italy in the 12 months before and after COVID-19 containment measures implementation by diagnosis class.. Pedianet 2019–2021, Table S5: Diagnosis classes with ICD9-CM codes and descriptive diagnosis.
