# Supplementary material for: Antibiotic Prescription Patterns in the Paediatric Primary Care Setting before and after the COVID-19 Pandemic in Italy: An Analysis Using the AWaRe Metrics
Source: Antibiotics (Basel). 2022 Mar 29;11(4):457. doi: 10.3390/antibiotics11040457 (PMC9025823; doi:10.3390/antibiotics11040457)
Supplement: Supplementary file 1 [file antibiotics-11-00457-s001.zip › antibiotics-1641250-supplementary.pdf]

Supplementary material

Table S1. Demographic characteristics of children included in the analysis in the 12 months before and after COVID-19 containment measures implementation. Pedianet 2019-2021)

Table S2. Monthly antibiotic prescriptions in children 0-14 years of age in Italy in the 12 months before and after COVID-19 containment measures implementation. Pedianet 2019-2021)

Figure S1. Interrupted time series of the monthly index of telemedicine visits (black) and well-child visits (gray) in children 0-14 years of age in Italy in the 12 months before and after COVID-19 containment measures implementation along with 95% CI. Pedianet 2019-2021

Table S3. Prevalence of. monthly antibiotic prescriptions in children 0-14 years of age in Italy in the 12 months before and after COVID-19 containment measures implementation by diagnosis class on total monthly antibiotic prescriptions. Pedianet 2019-2021

Table S4. Monthly antibiotic prescriptions in children 0-14 years of age in Italy in the 12 months before and after COVID-19 containment measures implementation by diagnosis class.. Pedianet 2019-2021

Table S5. Diagnosis classes with ICD9-CM codes and descriptive diagnosis.

Table S1. Demographic characteristics of children included in the analysis in the 12 months before and after COVID-19 containment measures implementation. Pedianet 2019-2021)

	Before COVID-19 containement measures implementation (N=150829)	After COVID-19 containement measures implementation (N=151067)
Sex, N(%)		
Female	72558 (48.1)	72609 (48.1)
Male	78271 (51.9)	78458 (51.9)
Age		
Mean (SD)	6.280 (4.096)	6.468 (4.116)
Range	0.000 - 13.980	0.000 - 13.980
Age class, N(%)		
<1 year	19616 (13.0)	18251 (12.1)
1-4 years	42890 (28.4)	41982 (27.8)
10-14 years	34370 (22.8)	36759 (24.3)
5-9 years	53953 (35.8)	54075 (35.8)
Region, N(%)		
Abruzzo	13664 (9.1)	13858 (9.2)
Campania	11279 (7.5)	11542 (7.6)
Friuli-Ven. Giulia	3464 (2.3)	3470 (2.3)
Lazio	6492 (4.3)	6649 (4.4)
Liguria	1148 (0.8)	1162 (0.8)
Lombardia	11620 (7.7)	11359 (7.5)
Marche	14972 (9.9)	15066 (10.0)
Piemonte	15183 (10.1)	15496 (10.3)
Puglia	122 (0.1)	404 (0.3)
Sardegna	3093 (2.1)	3148 (2.1)
Sicilia	10820 (7.2)	10869 (7.2)
Toscana	2044 (1.4)	1996 (1.3)
Veneto	56928 (37.7)	56048 (37.1)
Location, N(%)		
North	88343 (58.6)	87535 (57.9)
Centre	23508 (15.6)	23711 (15.7)
South with Islands	38978 (25.8)	39821 (26.4)

Table S2. Monthly antibiotic prescriptions in children 0-14 years of age in Italy in the 12 months before and after COVID-19 containment measures implementation.. Pedianet 2019-2021)

Period		Person-years	Total	Access				Watch	Other	Antibiotic index	Access to Watch index	Amoxicillin to Co-amoxiclav index
				Amoxicillin	Co-amoxiclav	Other Access	Total					
		N	N	N (%) on total ab	N (%) on total ab	N (%) on total ab	N (%) on total ab	N (%) on total ab	N (%) on total ab	Index	Index	Index
Before COVID-19 containement measures implementation	Mar-Apr 2019	11158	11614	2566 (22.1)	4028 (34.7)	125 (1.1)	6719 (57.9)	4845 (41.7)	50 (0.4)	1.04	1.39	0.64
	Apr-May 2019	10856	7797	1716 (22)	2606 (33.4)	67 (0.9)	4389 (56.3)	3396 (43.6)	12 (0.2)	0.72	1.29	0.66
	May-Jun 2019	11278	8596	2032 (23.6)	2861 (33.3)	94 (1.1)	4987 (58)	3591 (41.8)	18 (0.2)	0.76	1.39	0.71
	Jun-Jul 2019	10973	6504	1445 (22.2)	2104 (32.3)	41 (0.6)	3590 (55.2)	2903 (44.6)	11 (0.2)	0.59	1.24	0.69
	Jul-Aug 2019	11407	4430	845 (19.1)	1631 (36.8)	46 (1)	2522 (56.9)	1894 (42.8)	14 (0.3)	0.39	1.33	0.52
	Aug-Sep 2019	11470	3049	448 (14.7)	1130 (37.1)	42 (1.4)	1620 (53.1)	1421 (46.6)	8 (0.3)	0.27	1.14	0.4
	Sep-Oct 2019	11169	5359	1220 (22.8)	1787 (33.3)	72 (1.3)	3079 (57.5)	2260 (42.2)	20 (0.4)	0.48	1.36	0.68
	Oct-Nov 2019	11613	7445	1789 (24)	2401 (32.2)	83 (1.1)	4273 (57.4)	3154 (42.4)	18 (0.2)	0.64	1.35	0.75
	Nov-Dec 2019	11297	9767	2266 (23.2)	3236 (33.1)	100 (1)	5602 (57.4)	4141 (42.4)	24 (0.3)	0.86	1.35	0.7
	Dec 2019 - Jan 2020	11740	9044	2254 (24.9)	2873 (31.8)	107 (1.2)	5234 (57.9)	3788 (41.9)	22 (0.2)	0.77	1.38	0.78
	Jan-Feb 2020	11797	13697	3049 (22.3)	4511 (32.9)	137 (1)	7697 (56.2)	5975 (43.6)	25 (0.2)	1.16	1.29	0.68
	Feb-Mar 2020	11075	10401	2078 (20)	3649 (35.1)	119 (1.1)	5846 (56.2)	4541 (43.7)	14 (0.1)	0.94	1.29	0.57
After COVID-19 containement measures implementation	Mar-Apr 2020	11868	2244	370 (16.5)	833 (37.1)	27 (1.2)	1230 (54.8)	1011 (45.1)	3 (0.1)	0.19	1.22	0.44
	Apr-May 2020	11514	1304	210 (16.1)	510 (39.1)	18 (1.4)	738 (56.6)	565 (43.3)	1 (0.1)	0.11	1.31	0.41
	May-Jun 2020	11935	1357	212 (15.6)	581 (42.8)	27 (2)	820 (60.4)	536 (39.5)	1 (0.1)	0.11	1.53	0.36
	Jun-Jul 2020	11592	1445	241 (16.7)	646 (44.7)	16 (1.1)	903 (62.5)	541 (37.4)	1 (0.1)	0.12	1.67	0.37
	Jul-Aug 2020	12026	1948	327 (16.8)	814 (41.8)	21 (1.1)	1162 (59.7)	783 (40.2)	3 (0.2)	0.16	1.48	0.4
	Aug-Sep 2020	12088	2144	316 (14.7)	904 (42.2)	25 (1.2)	1245 (58.1)	886 (41.3)	13 (0.6)	0.18	1.41	0.35
	Sep-Oct 2020	11775	2176	369 (17)	806 (37)	49 (2.3)	1224 (56.3)	938 (43.1)	14 (0.6)	0.18	1.3	0.46
	Oct-Nov 2020	12249	2827	541 (19.1)	968 (34.2)	45 (1.6)	1554 (55)	1262 (44.6)	11 (0.4)	0.23	1.23	0.56
	Nov-Dec 2020	11917	2103	410 (19.5)	723 (34.4)	35 (1.7)	1168 (55.5)	933 (44.4)	2 (0.1)	0.18	1.25	0.57
	Dec 2020 - Jan 2021	12373	1458	287 (19.7)	556 (38.1)	29 (2)	872 (59.8)	586 (40.2)	0 (0)	0.12	1.49	0.52
	Jan-Feb 2021	12447	2148	386 (18)	846 (39.4)	29 (1.4)	1261 (58.7)	887 (41.3)	0 (0)	0.17	1.42	0.46
	Feb-Mar 2021	11292	2124	424 (20)	787 (37.1)	28 (1.3)	1239 (58.3)	885 (41.7)	0 (0)	0.19	1.4	0.54

Figure S1. Interrupted time series of the monthly index of telemedicine visits (black) and well-child visits (gray) in children 0-14 years of age in Italy in the 12 months before and after COVID-19 containment measures implementation along with 95% CI. Pedianet 2019-2021

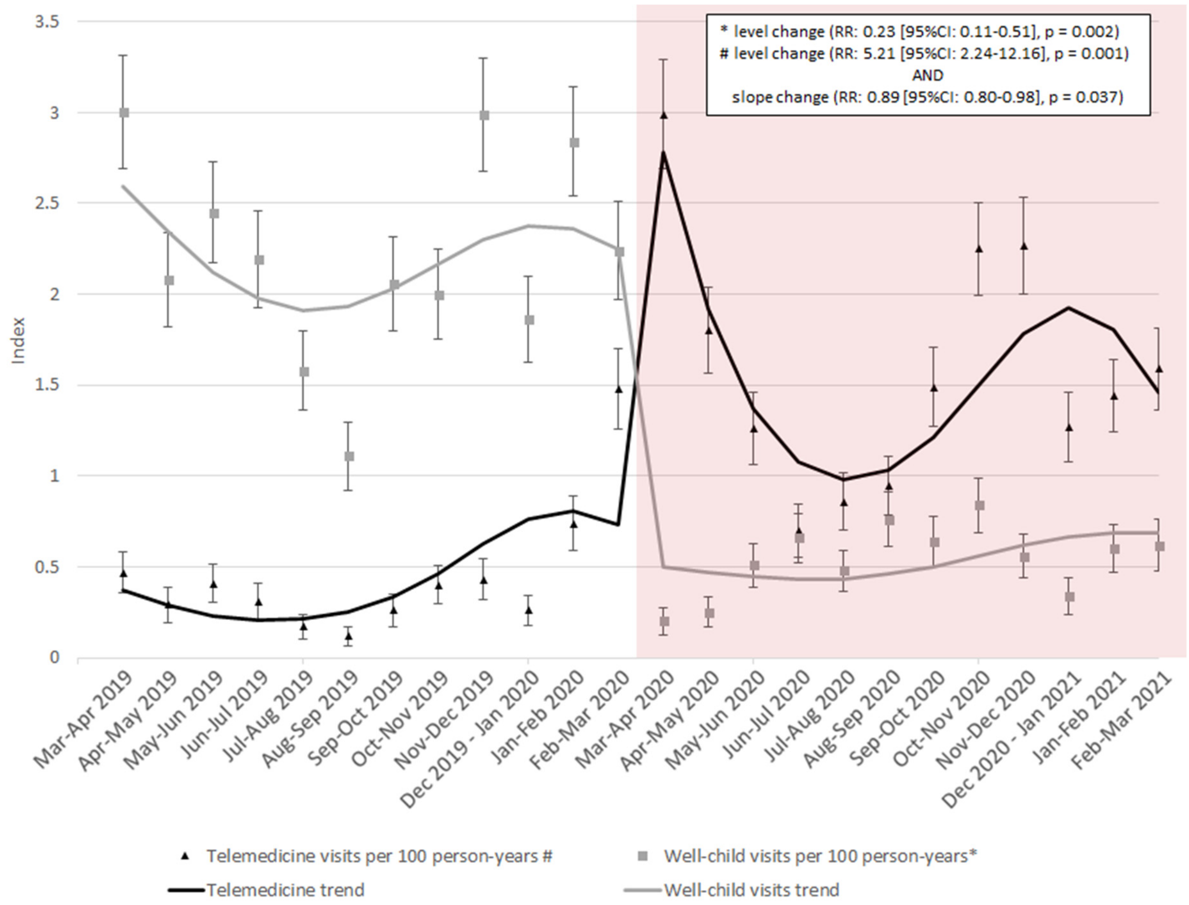

Table S3. Prevalence of. monthly antibiotic prescriptions in children 0-14 years of age in Italy in the 12 months before and after COVID-19 containment measures implementation by diagnosis class on total monthly antibiotic prescriptions. Pedianet 2019-2021

Period		LRTI<br><br>N (%) on total ab	SSTI<br><br>N (%) on total ab	URTI<br><br>N (%) on total ab	UTI<br><br>N (%) on total ab	NA<br><br>N (%) on total ab
Before COVID-19 containement measures implementation	Mar-Apr 2019	2077 (17.9)	199 (1.7)	6152 (53)	124 (1.1)	3062 (26.4)
	Apr-May 2019	1239 (15.9)	168 (2.2)	4279 (54.9)	114 (1.5)	1997 (25.6)
	May-Jun 2019	1253 (14.6)	191 (2.2)	4852 (56.4)	96 (1.1)	2204 (25.6)
	Jun-Jul 2019	959 (14.7)	209 (3.2)	3355 (51.6)	88 (1.4)	1893 (29.1)
	Jul-Aug 2019	550 (12.4)	221 (5)	2153 (48.6)	94 (2.1)	1412 (31.9)
	Aug-Sep 2019	419 (13.7)	227 (7.5)	1375 (45.1)	75 (2.5)	953 (31.3)
	Sep-Oct 2019	813 (15.2)	212 (4)	2810 (52.4)	117 (2.2)	1408 (26.3)
	Oct-Nov 2019	1222 (16.4)	168 (2.3)	4060 (54.5)	110 (1.5)	1885 (25.3)
	Nov-Dec 2019	1739 (17.8)	192 (2)	5343 (54.7)	136 (1.4)	2357 (24.1)
	Dec 2019 - Jan 2020	2064 (22.8)	176 (2)	4609 (51)	101 (1.1)	2094 (23.2)
	Jan-Feb 2020	3624 (26.5)	188 (1.4)	6529 (47.7)	119 (0.9)	3237 (23.6)
	Feb-Mar 2020	2555 (24.6)	141 (1.4)	5016 (48.2)	114 (1.1)	2575 (24.8)
After COVID-19 containement measures implementation	Mar-Apr 2020	285 (12.7)	126 (5.6)	982 (43.8)	100 (4.5)	751 (33.5)
	Apr-May 2020	124 (9.5)	120 (9.2)	526 (40.3)	91 (7)	443 (34)
	May-Jun 2020	85 (6.3)	157 (11.6)	552 (40.7)	115 (8.5)	448 (33)
	Jun-Jul 2020	75 (5.2)	148 (10.2)	624 (43.2)	98 (6.8)	500 (34.6)
	Jul-Aug 2020	158 (8.1)	147 (7.6)	880 (45.2)	84 (4.3)	679 (34.9)
	Aug-Sep 2020	127 (5.9)	154 (7.2)	748 (34.9)	68 (3.2)	1047 (48.8)
	Sep-Oct 2020	218 (10)	121 (5.6)	665 (30.6)	59 (2.7)	1113 (51.2)
	Oct-Nov 2020	309 (10.9)	81 (2.9)	886 (31.3)	84 (3)	1467 (51.9)
	Nov-Dec 2020	145 (6.9)	100 (4.8)	648 (30.8)	85 (4)	1125 (53.5)
	Dec 2020 - Jan 2021	80 (5.5)	56 (3.8)	390 (26.8)	74 (5.1)	858 (58.9)
	Jan-Feb 2021	110 (5.1)	98 (4.6)	657 (30.6)	95 (4.4)	1188 (55.3)
	Feb-Mar 2021	127 (6)	104 (4.9)	702 (33.1)	61 (2.9)	1130 (53.2)

Table S4. Monthly antibiotic prescriptions in children 0-14 years of age in Italy in the 12 months before and after COVID-19 containment measures implementation by diagnosis.. Pedianet 2019-2021

Class	Period		Total	Access				Watch	Other	Antibiotic index	Access to Watch index	Amoxicillin to Co-amoxiclav index
				Amoxicillin	Co-amoxiclav	Other Access	Total					
			N	N (%) on total ab	N (%) on total ab	N (%) on total ab	N (%) on total ab	N (%) on total ab	N (%) on total ab	Index	Index	Index
LRTI	*Before COVID-19 containement measures implementation*	Mar-Apr 2019	2077	315 (15.2)	551 (26.5)	6 (0.3)	872 (42)	1156 (55.7)	49 (2.4)	0.19	0.75	0.57
		Apr-May 2019	1239	189 (15.3)	292 (23.6)	10 (0.8)	491 (39.6)	737 (59.5)	11 (0.9)	0.11	0.67	0.65
		May-Jun 2019	1253	184 (14.7)	288 (23)	9 (0.7)	481 (38.4)	757 (60.4)	15 (1.2)	0.11	0.64	0.64
		Jun-Jul 2019	959	122 (12.7)	181 (18.9)	4 (0.4)	307 (32)	643 (67)	9 (0.9)	0.09	0.48	0.67
		Jul-Aug 2019	550	63 (11.5)	118 (21.5)	3 (0.5)	184 (33.5)	354 (64.4)	12 (2.2)	0.05	0.52	0.53
		Aug-Sep 2019	419	48 (11.5)	88 (21)	1 (0.2)	137 (32.7)	277 (66.1)	5 (1.2)	0.04	0.49	0.55
		Sep-Oct 2019	813	117 (14.4)	170 (20.9)	4 (0.5)	291 (35.8)	506 (62.2)	16 (2)	0.07	0.58	0.69
		Oct-Nov 2019	1222	200 (16.4)	295 (24.1)	5 (0.4)	500 (40.9)	710 (58.1)	12 (1)	0.11	0.7	0.68
		Nov-Dec 2019	1739	330 (19)	397 (22.8)	10 (0.6)	737 (42.4)	981 (56.4)	21 (1.2)	0.15	0.75	0.83
		Dec 2019 - Jan 2020	2064	445 (21.6)	475 (23)	10 (0.5)	930 (45.1)	1114 (54)	20 (1)	0.18	0.83	0.94
		Jan-Feb 2020	3624	790 (21.8)	966 (26.7)	23 (0.6)	1779 (49.1)	1823 (50.3)	22 (0.6)	0.31	0.98	0.82
		Feb-Mar 2020	2555	494 (19.3)	682 (26.7)	18 (0.7)	1194 (46.7)	1347 (52.7)	14 (0.5)	0.23	0.89	0.72
	After COVID-19 containement measures implementation	Mar-Apr 2020	285	34 (11.9)	68 (23.9)	4 (1.4)	106 (37.2)	177 (62.1)	2 (0.7)	0.02	0.6	0.5
		Apr-May 2020	124	11 (8.9)	33 (26.6)	0 (0)	44 (35.5)	79 (63.7)	1 (0.8)	0.01	0.56	0.33
		May-Jun 2020	85	7 (8.2)	21 (24.7)	1 (1.2)	29 (34.1)	55 (64.7)	1 (1.2)	0.01	0.53	0.33
		Jun-Jul 2020	75	11 (14.7)	23 (30.7)	0 (0)	34 (45.3)	40 (53.3)	1 (1.3)	0.01	0.85	0.48
		Jul-Aug 2020	158	10 (6.3)	33 (20.9)	1 (0.6)	44 (27.8)	112 (70.9)	2 (1.3)	0.01	0.39	0.3
		Aug-Sep 2020	127	10 (7.9)	20 (15.7)	0 (0)	30 (23.6)	87 (68.5)	10 (7.9)	0.01	0.34	0.5
		Sep-Oct 2020	218	18 (8.3)	42 (19.3)	1 (0.5)	61 (28)	144 (66.1)	13 (6)	0.02	0.42	0.43
		Oct-Nov 2020	309	33 (10.7)	55 (17.8)	0 (0)	88 (28.5)	211 (68.3)	10 (3.2)	0.03	0.42	0.6
		Nov-Dec 2020	145	14 (9.7)	24 (16.6)	0 (0)	38 (26.2)	105 (72.4)	2 (1.4)	0.01	0.36	0.58
		Dec 2020 - Jan 2021	80	7 (8.8)	13 (16.3)	0 (0)	20 (25)	60 (75)	0 (0)	0.01	0.33	0.54
		Jan-Feb 2021	110	16 (14.5)	18 (16.4)	1 (0.9)	35 (31.8)	75 (68.2)	0 (0)	0.01	0.47	0.89
		Feb-Mar 2021	127	9 (7.1)	29 (22.8)	1 (0.8)	39 (30.7)	88 (69.3)	0 (0)	0.01	0.44	0.31
SSTI	*Before COVID-19 containement measures implementation*	Mar-Apr 2019	199	43 (21.6)	91 (45.7)	1 (0.5)	135 (67.8)	64 (32.2)	0 (0)	0.02	2.11	0.47
		Apr-May 2019	168	27 (16.1)	92 (54.8)	2 (1.2)	121 (72)	47 (28)	0 (0)	0.02	2.57	0.29
		May-Jun 2019	191	34 (17.8)	98 (51.3)	2 (1)	134 (70.2)	57 (29.8)	0 (0)	0.02	2.35	0.35
		Jun-Jul 2019	209	23 (11)	112 (53.6)	1 (0.5)	136 (65.1)	73 (34.9)	0 (0)	0.02	1.86	0.21
		Jul-Aug 2019	221	31 (14)	124 (56.1)	1 (0.5)	156 (70.6)	65 (29.4)	0 (0)	0.02	2.4	0.25
		Aug-Sep 2019	227	21 (9.3)	144 (63.4)	0 (0)	165 (72.7)	62 (27.3)	0 (0)	0.02	2.66	0.15
		Sep-Oct 2019	212	26 (12.3)	127 (59.9)	2 (0.9)	155 (73.1)	57 (26.9)	0 (0)	0.02	2.72	0.2
		Oct-Nov 2019	168	21 (12.5)	95 (56.5)	2 (1.2)	118 (70.2)	50 (29.8)	0 (0)	0.01	2.36	0.22
		Nov-Dec 2019	192	36 (18.8)	106 (55.2)	2 (1)	144 (75)	48 (25)	0 (0)	0.02	3	0.34
		Dec 2019 - Jan 2020	176	31 (17.6)	90 (51.1)	3 (1.7)	124 (70.5)	51 (29)	1 (0.6)	0.01	2.43	0.34

	After COVID-19 containement measures implementation	Jan-Feb 2020	188	37 (19.7)	96 (51.1)	4 (2.1)	137 (72.9)	51 (27.1)	0 (0)	0.02	2.69	0.39
		Feb-Mar 2020	141	19 (13.5)	88 (62.4)	2 (1.4)	109 (77.3)	32 (22.7)	0 (0)	0.01	3.41	0.22
		Mar-Apr 2020	126	24 (19)	65 (51.6)	1 (0.8)	90 (71.4)	36 (28.6)	0 (0)	0.01	2.5	0.37
		Apr-May 2020	120	19 (15.8)	72 (60)	0 (0)	91 (75.8)	29 (24.2)	0 (0)	0.01	3.14	0.26
		May-Jun 2020	157	27 (17.2)	84 (53.5)	3 (1.9)	114 (72.6)	43 (27.4)	0 (0)	0.01	2.65	0.32
		Jun-Jul 2020	148	22 (14.9)	89 (60.1)	1 (0.7)	112 (75.7)	36 (24.3)	0 (0)	0.01	3.11	0.25
		Jul-Aug 2020	147	22 (15)	81 (55.1)	2 (1.4)	105 (71.4)	41 (27.9)	1 (0.7)	0.01	2.56	0.27
		Aug-Sep 2020	154	22 (14.3)	93 (60.4)	2 (1.3)	117 (76)	37 (24)	0 (0)	0.01	3.16	0.24
		Sep-Oct 2020	121	15 (12.4)	66 (54.5)	3 (2.5)	84 (69.4)	37 (30.6)	0 (0)	0.01	2.27	0.23
		Oct-Nov 2020	81	12 (14.8)	50 (61.7)	0 (0)	62 (76.5)	19 (23.5)	0 (0)	0.01	3.26	0.24
		Nov-Dec 2020	100	12 (12)	55 (55)	0 (0)	67 (67)	33 (33)	0 (0)	0.01	2.03	0.22
		Dec 2020 - Jan 2021	56	13 (23.2)	21 (37.5)	0 (0)	34 (60.7)	22 (39.3)	0 (0)	0	1.55	0.62
		Jan-Feb 2021	98	16 (16.3)	59 (60.2)	0 (0)	75 (76.5)	23 (23.5)	0 (0)	0.01	3.26	0.27
		Feb-Mar 2021	104	19 (18.3)	62 (59.6)	1 (1)	82 (78.8)	22 (21.2)	0 (0)	0.01	3.73	0.31
URTI	*Before COVID-19 containement measures implementation*	Mar-Apr 2019	6152	1757 (28.6)	2092 (34)	74 (1.2)	3923 (63.8)	2228 (36.2)	1 (0)	0.55	1.76	0.84
		Apr-May 2019	4279	1202 (28.1)	1381 (32.3)	31 (0.7)	2614 (61.1)	1664 (38.9)	1 (0)	0.39	1.57	0.87
		May-Jun 2019	4852	1433 (29.5)	1575 (32.5)	42 (0.9)	3050 (62.9)	1799 (37.1)	3 (0.1)	0.43	1.7	0.91
		Jun-Jul 2019	3355	988 (29.4)	1060 (31.6)	20 (0.6)	2068 (61.6)	1285 (38.3)	2 (0.1)	0.31	1.61	0.93
		Jul-Aug 2019	2153	525 (24.4)	741 (34.4)	17 (0.8)	1283 (59.6)	868 (40.3)	2 (0.1)	0.19	1.48	0.71
		Aug-Sep 2019	1375	257 (18.7)	456 (33.2)	16 (1.2)	729 (53)	643 (46.8)	3 (0.2)	0.12	1.13	0.56
		Sep-Oct 2019	2809	826 (29.4)	871 (31)	41 (1.5)	1738 (61.9)	1070 (38.1)	1 (0)	0.25	1.62	0.95
		Oct-Nov 2019	4060	1210 (29.8)	1271 (31.3)	37 (0.9)	2518 (62)	1537 (37.9)	5 (0.1)	0.35	1.64	0.95
		Nov-Dec 2019	5343	1492 (27.9)	1765 (33)	56 (1)	3313 (62)	2029 (38)	1 (0)	0.47	1.63	0.85
		Dec 2019 - Jan 2020	4609	1356 (29.4)	1458 (31.6)	55 (1.2)	2869 (62.2)	1740 (37.8)	0 (0)	0.39	1.65	0.93
		Jan-Feb 2020	6529	1669 (25.6)	2105 (32.2)	80 (1.2)	3854 (59)	2674 (41)	1 (0)	0.55	1.44	0.79
		Feb-Mar 2020	5016	1148 (22.9)	1821 (36.3)	67 (1.3)	3036 (60.5)	1980 (39.5)	0 (0)	0.45	1.53	0.63
	After COVID-19 containement measures implementation	Mar-Apr 2020	982	191 (19.5)	363 (37)	11 (1.1)	565 (57.5)	417 (42.5)	0 (0)	0.08	1.35	0.53
		Apr-May 2020	526	113 (21.5)	200 (38)	5 (1)	318 (60.5)	208 (39.5)	0 (0)	0.05	1.53	0.56
		May-Jun 2020	552	107 (19.4)	225 (40.8)	9 (1.6)	341 (61.8)	211 (38.2)	0 (0)	0.05	1.62	0.48
		Jun-Jul 2020	624	117 (18.8)	255 (40.9)	5 (0.8)	377 (60.4)	247 (39.6)	0 (0)	0.05	1.53	0.46
		Jul-Aug 2020	880	197 (22.4)	333 (37.8)	3 (0.3)	533 (60.6)	347 (39.4)	0 (0)	0.07	1.54	0.59
		Aug-Sep 2020	748	133 (17.8)	303 (40.5)	4 (0.5)	440 (58.8)	305 (40.8)	3 (0.4)	0.06	1.44	0.44
		Sep-Oct 2020	665	173 (26)	219 (32.9)	10 (1.5)	402 (60.5)	263 (39.5)	0 (0)	0.06	1.53	0.79
		Oct-Nov 2020	886	267 (30.1)	293 (33.1)	5 (0.6)	565 (63.8)	321 (36.2)	0 (0)	0.07	1.76	0.91
		Nov-Dec 2020	648	176 (27.2)	215 (33.2)	10 (1.5)	401 (61.9)	247 (38.1)	0 (0)	0.05	1.62	0.82
		Dec 2020 - Jan 2021	390	116 (29.7)	125 (32.1)	6 (1.5)	247 (63.3)	143 (36.7)	0 (0)	0.03	1.73	0.93
		Jan-Feb 2021	657	164 (25)	235 (35.8)	10 (1.5)	409 (62.3)	248 (37.7)	0 (0)	0.05	1.65	0.7
		Feb-Mar 2021	702	173 (24.6)	253 (36)	7 (1)	433 (61.7)	269 (38.3)	0 (0)	0.06	1.61	0.68
UTI	*Before COVID-19 containement measures implementation*	Mar-Apr 2019	124	7 (5.6)	47 (37.9)	4 (3.2)	58 (46.8)	66 (53.2)	0 (0)	0.01	0.88	0.15
		Apr-May 2019	114	5 (4.4)	43 (37.7)	2 (1.8)	50 (43.9)	64 (56.1)	0 (0)	0.01	0.78	0.12
		May-Jun 2019	96	7 (7.3)	33 (34.4)	5 (5.2)	45 (46.9)	51 (53.1)	0 (0)	0.01	0.88	0.21
		Jun-Jul 2019	88	6 (6.8)	24 (27.3)	2 (2.3)	32 (36.4)	56 (63.6)	0 (0)	0.01	0.57	0.25
		Jul-Aug 2019	94	5 (5.3)	34 (36.2)	5 (5.3)	44 (46.8)	50 (53.2)	0 (0)	0.01	0.88	0.15
		Aug-Sep 2019	75	0 (0)	20 (26.7)	2 (2.7)	22 (29.3)	53 (70.7)	0 (0)	0.01	0.42	0
		Sep-Oct 2019	117	7 (6)	32 (27.4)	5 (4.3)	44 (37.6)	73 (62.4)	0 (0)	0.01	0.6	0.22
		Oct-Nov 2019	110	7 (6.4)	50 (45.5)	2 (1.8)	59 (53.6)	51 (46.4)	0 (0)	0.01	1.16	0.14

	After COVID-19 containement measures implementation	Nov-Dec 2019	136	10 (7.4)	53 (39)	3 (2.2)	66 (48.5)	70 (51.5)	0 (0)	0.01	0.94	0.19
		Dec 2019 - Jan 2020	101	5 (5)	47 (46.5)	2 (2)	54 (53.5)	47 (46.5)	0 (0)	0.01	1.15	0.11
		Jan-Feb 2020	119	8 (6.7)	42 (35.3)	2 (1.7)	52 (43.7)	67 (56.3)	0 (0)	0.01	0.78	0.19
		Feb-Mar 2020	114	5 (4.4)	44 (38.6)	4 (3.5)	53 (46.5)	61 (53.5)	0 (0)	0.01	0.87	0.11
		Mar-Apr 2020	100	1 (1)	34 (34)	2 (2)	37 (37)	63 (63)	0 (0)	0.01	0.59	0.03
		Apr-May 2020	91	0 (0)	38 (41.8)	1 (1.1)	39 (42.9)	52 (57.1)	0 (0)	0.01	0.75	0
		May-Jun 2020	115	4 (3.5)	49 (42.6)	5 (4.3)	58 (50.4)	57 (49.6)	0 (0)	0.01	1.02	0.08
		Jun-Jul 2020	98	2 (2)	46 (46.9)	3 (3.1)	51 (52)	47 (48)	0 (0)	0.01	1.09	0.04
		Jul-Aug 2020	84	3 (3.6)	35 (41.7)	3 (3.6)	41 (48.8)	43 (51.2)	0 (0)	0.01	0.95	0.09
		Aug-Sep 2020	68	1 (1.5)	31 (45.6)	1 (1.5)	33 (48.5)	35 (51.5)	0 (0)	0.01	0.94	0.03
		Sep-Oct 2020	59	2 (3.4)	22 (37.3)	1 (1.7)	25 (42.4)	34 (57.6)	0 (0)	0.01	0.74	0.09
		Oct-Nov 2020	84	4 (4.8)	40 (47.6)	3 (3.6)	47 (56)	36 (42.9)	1 (1.2)	0.01	1.31	0.1
		Nov-Dec 2020	85	4 (4.7)	33 (38.8)	0 (0)	37 (43.5)	48 (56.5)	0 (0)	0.01	0.77	0.12
		Dec 2020 - Jan 2021	74	5 (6.8)	27 (36.5)	1 (1.4)	33 (44.6)	41 (55.4)	0 (0)	0.01	0.8	0.19
		Jan-Feb 2021	95	1 (1.1)	44 (46.3)	2 (2.1)	47 (49.5)	48 (50.5)	0 (0)	0.01	0.98	0.02
		Feb-Mar 2021	61	1 (1.6)	27 (44.3)	3 (4.9)	31 (50.8)	30 (49.2)	0 (0)	0.01	1.03	0.04
NA	*Before COVID-19 containement measures implementation*	Mar-Apr 2019	3062	444 (14.5)	1247 (40.7)	40 (1.3)	1731 (56.5)	1331 (43.5)	0 (0)	0.27	1.3	0.36
		Apr-May 2019	1997	293 (14.7)	798 (40)	22 (1.1)	1113 (55.7)	884 (44.3)	0 (0)	0.18	1.26	0.37
		May-Jun 2019	2204	374 (17)	867 (39.3)	36 (1.6)	1277 (57.9)	927 (42.1)	0 (0)	0.2	1.38	0.43
		Jun-Jul 2019	1893	306 (16.2)	727 (38.4)	14 (0.7)	1047 (55.3)	846 (44.7)	0 (0)	0.17	1.24	0.42
		Jul-Aug 2019	1412	221 (15.7)	614 (43.5)	20 (1.4)	855 (60.6)	557 (39.4)	0 (0)	0.12	1.54	0.36
		Aug-Sep 2019	953	122 (12.8)	422 (44.3)	23 (2.4)	567 (59.5)	386 (40.5)	0 (0)	0.08	1.47	0.29
		Sep-Oct 2019	1408	244 (17.3)	587 (41.7)	20 (1.4)	851 (60.4)	554 (39.3)	3 (0.2)	0.13	1.54	0.42
		Oct-Nov 2019	1885	351 (18.6)	690 (36.6)	37 (2)	1078 (57.2)	806 (42.8)	1 (0.1)	0.16	1.34	0.51
		Nov-Dec 2019	2357	398 (16.9)	915 (38.8)	29 (1.2)	1342 (56.9)	1013 (43)	2 (0.1)	0.21	1.32	0.43
		Dec 2019 - Jan 2020	2094	417 (19.9)	803 (38.3)	37 (1.8)	1257 (60)	836 (39.9)	1 (0)	0.18	1.5	0.52
		Jan-Feb 2020	3237	545 (16.8)	1302 (40.2)	28 (0.9)	1875 (57.9)	1360 (42)	2 (0.1)	0.27	1.38	0.42
		Feb-Mar 2020	2575	412 (16)	1014 (39.4)	28 (1.1)	1454 (56.5)	1121 (43.5)	0 (0)	0.23	1.3	0.41
	After COVID-19 containement measures implementation	Mar-Apr 2020	751	120 (16)	303 (40.3)	9 (1.2)	432 (57.5)	318 (42.3)	1 (0.1)	0.06	1.36	0.4
		Apr-May 2020	443	67 (15.1)	167 (37.7)	12 (2.7)	246 (55.5)	197 (44.5)	0 (0)	0.04	1.25	0.4
		May-Jun 2020	448	67 (15)	202 (45.1)	9 (2)	278 (62.1)	170 (37.9)	0 (0)	0.04	1.64	0.33
		Jun-Jul 2020	500	89 (17.8)	233 (46.6)	7 (1.4)	329 (65.8)	171 (34.2)	0 (0)	0.04	1.92	0.38
		Jul-Aug 2020	679	95 (14)	332 (48.9)	12 (1.8)	439 (64.7)	240 (35.3)	0 (0)	0.06	1.83	0.29
		Aug-Sep 2020	1047	150 (14.3)	457 (43.6)	18 (1.7)	625 (59.7)	422 (40.3)	0 (0)	0.09	1.48	0.33
		Sep-Oct 2020	1113	161 (14.5)	457 (41.1)	34 (3.1)	652 (58.6)	460 (41.3)	1 (0.1)	0.09	1.42	0.35
		Oct-Nov 2020	1467	225 (15.3)	530 (36.1)	37 (2.5)	792 (54)	675 (46)	0 (0)	0.12	1.17	0.42
		Nov-Dec 2020	1125	204 (18.1)	396 (35.2)	25 (2.2)	625 (55.6)	500 (44.4)	0 (0)	0.09	1.25	0.52
		Dec 2020 - Jan 2021	858	146 (17)	370 (43.1)	22 (2.6)	538 (62.7)	320 (37.3)	0 (0)	0.07	1.68	0.39
		Jan-Feb 2021	1188	189 (15.9)	490 (41.2)	16 (1.3)	695 (58.5)	493 (41.5)	0 (0)	0.1	1.41	0.39
		Feb-Mar 2021	1130	222 (19.6)	416 (36.8)	16 (1.4)	654 (57.9)	476 (42.1)	0 (0)	0.1	1.37	0.53

Diagnosis definition

Diagnostic categories for common outpatient infections are described in Table s4. All diagnoses linked to the same visit (max 3) were classified into these categories according to ICD-9CM code, free text, or descriptive diagnosis and then linked to the specific prescription. Free text diagnoses were manually classified by two investigators (EB and CL), and when not in agreement (less than 0.2% of cases), a consensus was achieved with a third investigator (DD).

Table S5. Diagnosis classes with ICD9-CM codes and descriptive diagnosis.

Diagnosis class	Diagnosis	ICD9-CM codes	Descriptive diagnosis (English)	Descriptive diagnosis (Italian)
URTI	Acute otitis media (AOM)	381, 382	Acute suppurative otitis media, Acute non-suppurative otitis media	otite media acuta con perforazione, otite media acuta, otite media essudativa
	Sinusitis	461, 473	sinusitis, chronic sinusitis	sinusite acuta e cronica
	Pharyngitis	034, 462, 463	acute pharyngitis, acute tonsillitis, streptococcal sore throat and scarlet fever	tonsillite, faringotonsillite, faringite, scarlattina
	Upper respiratory tract infection (URTI)	465	laryngitis, tracheitis, upper respiratory tract infections	laringite, tracheite, infezioni delle alte vie respiratorie
LRTI	Bronchitis/bronchiolitis	466, 490, 491, 496	acute bronchitis and bronchiolitis, chronic bronchitis, chronic airway obstruction	bronchite acute, bronchiolite acuta, bronchite cronica, bronco pneumopatia cronico ostruttiva
	Pneumonia	481, 482, 483, 484, 485, 486	pneumonia, bronchopneumonia	polmonite, broncopolmonite
	Lower respiratory tract infection (LRTI)	NA	lower respiratory tract infections (unspecified)	infezioni delle basse vie respiratorie (non specificato)
UTI	Urinary tract infection (UTI)	590.1, 590.2, 590.8, 590.9, 595.0, 595.9, 599.0	acute pyelonephritis, renal abscess, other pyelonephritis/pyelonephritis, kidney infection (unspecified), acute cystitis, cystitis (unspecified), UTI (unspecified)	pielonefrite acuta, ascesso renale, infezione delle vie urinarie (non specificato), cistite (non specificato)
SSTI	Skin and Mucus Membranes Infection (SMMI)	110-111, 771.5, 611, 380.0, 035, 706, 684, 384, 372.1, 372.2, 522.4, 522.5, 027, 681, 685	Various infection of the skin and mucus membranes including skin wound, impetigo, dermatophytosis and dermatomycosis, folliculitis, infective otitis externa, myringitis, acne, dental/gingival abscess, erysipelas, conjunctivitis, bacterial zoonotic disease, animal bite,	ferita, impetigine, infezione della pelle, infezione dei tessuti molli, morso/graffio di animale, erisipela, dermatofitiosis, follicolite, otite esterna, miringite, anite streptococcica, acne, mastite, ascesso dentale/gengivale.

			cellulitis and abscess of finger and toe, pilonidal cyst	
	Soft tissue infection (STI)	682, 683, 475, 478.2, 728.86, 728.0	cellulitis, acute lymphadenitis, cutaneous abscess, peritonsillar and pharyngeal abscess, myositis, necrotizing fasciitis	cellulite, linfadenite, ascesso cutaneo, ascesso pertonsillare/retrofaringeo, miosite, fascite necrotizzante
